# Conformational Features of Topologically Classified RNA Secondary Structures

**DOI:** 10.1371/journal.pone.0039907

**Published:** 2012-07-05

**Authors:** Jimmy Ka Ho Chiu, Yi-Ping Phoebe Chen

**Affiliations:** Department of Computer Science and Computer Engineering, La Trobe University, Melbourne, Victoria, Australia; University of Lethbridge, Canada

## Abstract

**Background:**

Current RNA secondary structure prediction approaches predict prevalent pseudoknots such as the H-pseudoknot and kissing hairpin. The number of possible structures increases drastically when more complex pseudoknots are considered, thus leading to computational limitations. On the other hand, the enormous population of possible structures means not all of them appear in real RNA molecules. Therefore, it is of interest to understand how many of them really exist and the reasons for their preferred existence over the others, as any new findings revealed by this study might enhance the capability of future structure prediction algorithms for more accurate prediction of complex pseudoknots.

**Methodology/Principal Findings:**

A novel algorithm was devised to estimate the exact number of structural possibilities for a pseudoknot constructed with a specified number of base pair stems. Then, topological classification was applied to classify RNA pseudoknotted structures from data in the RNA STRAND database. By showing the vast possibilities and the real population, it is clear that most of these plausible complex pseudoknots are not observed. Moreover, from these classified motifs that exist in nature, some features were identified for further investigation. It was found that some features are related to helical stacking. Other features are still left open to discover underlying tertiary interactions.

**Conclusions:**

Results from topological classification suggest that complex pseudoknots are usually some well-known motifs that are themselves complex or the interaction results of some special motifs. Heuristics can be proposed to predict the essential parts of these complex motifs, even if the required thermodynamic parameters are currently unknown.

## Introduction

In additional to protein encoding, RNAs have been discovered to have various regulatory and catalytic roles in many biological processes [1]. RNAs with these roles are called non-coding RNAs (ncRNAs). In eukaryotes, microRNAs (miRNAs) are believed to act as an agent for transcriptional induction or repression, as well as translational silencing and messenger RNA (mRNA) degradation [2]. Similar functions have also been found in prokaryotes, and even performing translation stimulation and mRNA stabilization [3]. For example, in E. coli, RpoS translation is stimulated by DsrA under low temperature or by RprA under stress on the cell surface, whereas OxyS can repress RpoS translation under oxidative shock [4].

Studies also revealed the relationship between the structure of an RNA sequence and the functions of the RNAs [5], [6]. Thus, to predict the functions of a given RNA sequence, it becomes critical to correctly predict its structure. Moreover, it has been suggested that the RNA folding is hierarchical in a way that an RNA sequence itself determines its secondary structure which, in turn determines its tertiary structure [7]. Therefore, RNA secondary structure prediction is a very important problem since it helps in the determination of tertiary structure and function. Many proposed secondary structure prediction algorithms applied dynamic programming to compute the minimum free energy (MFE) secondary structure for a given RNA sequence. Mfold [8] is one of the earliest models that considers all possible pseudoknot-free structures in O(n^4^) time. Later, the dynamic programming approach was extended to include certain pseudoknot motifs [9]. Other approaches also emerged to predict particular types of pseudoknot motifs [10], [11]. Partition function is another mean expressed in some structure prediction strategies. It is used to estimate the base pairing probability of two specified nucleotides in a given RNA sequence, and hence the probability for every possible base pairs for pseudoknot-free structures [12]. Later, partition function calculation was also extended to include certain pseudoknots [13], [14]. Nonetheless, using a nearest neighbor interaction model, it has been proved that RNA secondary structure prediction with arbitrary pseudoknots is NP complete [15], [16]. Most of the current approaches predict the H-pseudoknot, the kissing hairpin and a few other pseudoknots. If more complex pseudoknots are going to be included, then it is necessary to select the most probable set from all possibilities. However, in the absence of relevant biological findings, it is difficult to determine what motifs are more favorable than the others.

**Figure 1:**
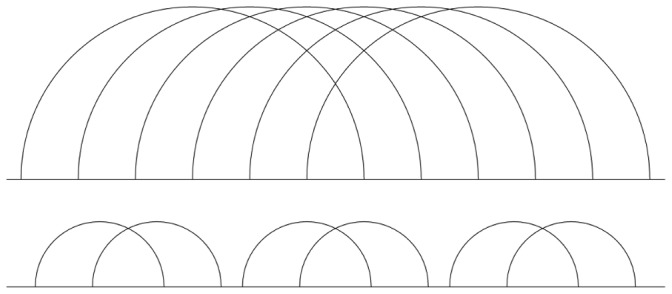
Two sample RNA secondary structures both having genus values  = 3. Both structures have their own genus equal to 3, but the lower one is a concatenation of three H-pseudoknots of genus equal to 1. The genus of the whole structure is the sum of the genera of its primitive components.

In this paper, we try to explore the existing complex pseudoknots (i.e. those other than the most prevalent ones such as the H-pseudoknot and the kissing hairpin) from real RNA sequences and seek some special structural features. For this purpose, it is necessary to classify arbitrary pseudoknots according to pre-defined complexity measures. One of the classification approaches is k-noncrossing matching [17], [18], where a k-noncrossing structure has no more than k –1 crossings of its base pair arcs (each arc representing a stem). Another approach is called k-partite, meaning that an RNA secondary structure can be divided into k substructures which are pseudoknot-free [19]. We are interested in another classification that applies quantum matrix field theory [20]. In this classification, an RNA secondary structure is analyzed to evaluate a quantity called genus. It is a non-negative integral value. If a secondary structure has a genus value of zero, then that structure must be pseudoknot-free, otherwise the value is positive. The definition of genus 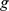 is given by 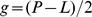, where 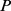 is the number of base pairs and 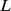 is the number of closed loops. The determination of 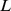 will be presented in the next section. The higher the genus value, the more complicated pseudoknotted substructure a secondary structure has, or the more pseudoknotted substructures it contains, or both. Figure 1 shows two distinct RNA secondary structures whose genera are both 3. Clearly, the upper structure is more complex than the lower one which concatenates three H-pseudoknots. In particular, the most prevalent pseudoknots such as the H-pseudoknot and the kissing hairpin both have their genera equal to one, meaning they are regarded as the simplest pseudoknots.

Since genus is an additive quantity, a secondary structure can be decomposed into numerous primitive components, and the genus of the original structure is the sum of the genera of its components [20]. These components are primitive in the sense that they are irreducible and non-nested within other substructures. The main reason for the decomposition is that, as mentioned, since a secondary structure may contain several pseudoknotted components or a single complicated pseudoknot to attain the same genus value, in order to fairly compare the complexity of pseudoknots that two arbitrary RNA secondary structures contain, it is necessary to extract their respective primitive components and compare the genera of these components instead of the whole structure. On the other hand, primitive components can also be viewed as building blocks because arbitrary secondary structures can be constructed with them. However, since the set of the building blocks is infinite, only certain secondary structures can be constructed with its limited subset. Using this perspective, a dynamic programming algorithm has been proposed to predict RNA secondary structure using a topological approach [21]. A secondary structure is called a γ-structure if the genus of each of its primitive components is at most γ. This algorithm incorporates 4 unique primitive components whose genera are all equal to 1 into the context-free grammar (CFG), and 2 of these 4 components are the most common H-pseudoknot and kissing hairpin. Therefore, the algorithm considers up to 1 structure. However, to expand the primitive component set, it was shown that it jumped from 4 unique structures for 1 structure to 3472 unique structures to be considered (Supplementary Material of [21]). The increase is even much larger for a higher order γ-structure.

It was shown that complex primitive pseudoknots (with genus >2) are very rare in nature [20]. In this work, we further illustrate this by comparing the number of distinct primitive pseudoknot motifs found with the population of equally complexity motifs. Furthermore, from these infrequent motifs we identify structural features, if any, which help explain their preferred existence over the rest of the population.

## Methods

We will first introduce the implementation basics for topological classification based on genus evaluation, and then our novel approach for primitive components enumeration will be shown. Finally, we will present the experimental details of our analysis.

As mentioned in the previous section, an RNA secondary structure is decomposed into primitive components and each component is then evaluated to obtain its genus value. The total genus of this secondary structure is the sum of the genera of its components. This general concept is already discussed in [20]. In this paper, we provide the implementation details of primitive component extraction and genus value evaluation.

The first step is to decompose the secondary structure into irreducible components. Using dot-parentheses notation, the structure is scanned from the beginning to discover any irreducible components. The pseudocode for irreducible component decomposition is as follows:

irr_start  = 1;

for i  = 1 to length of the structure sequence

s  =  structure symbol at position i;

if s is an opening bracket

increment the count for that bracket type;

else if s is a closing bracket

decrement the count for that bracket type;

if the counts for all the bracket types  = 0

extract the structure sequence from irr_start to i;

irr_start  =  i+1;

end if

end if

end for

**Figure 2:**
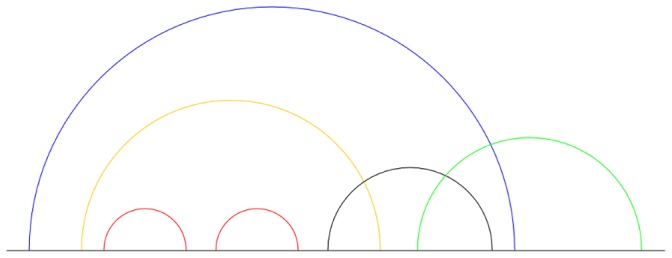
Extraction of nested base pairs. Starting from the leftmost base pair (blue arc), the determination of non-nested arcs is in the order of: blue, green, black and orange. The two nested base pairs (red arcs) are extracted as two irreducible components.

The second step involves the extraction of non-nested components from those irreducible components. By observing that the leftmost and the rightmost base pairs (note that they can be the same base pair) of any irreducible component are non-nested, any other base pairs that cross with them must also be non-nested. From these base pairs, another set of base pairs that cross with them are non-nested as well. This process is repeated until no more new non-nested base pairs are found. Figure 2 illustrates this process. Since the component is irreducible, all non-nested base pairs can be discovered. The pseudocode below shows this step:

non_nest_bp  =  Ø;

bp  =  leftmost base pair of irreducible component;

search_non_nest_bp(bp);

sub search_non_nest_bp (bp)

add bp to non_nest_bp;

pk  =  all base pairs crossing with bp;

for each basepair c in pk

if c is not in non_nest_bp

search_non_nest_bp(c);

end if

end for

end sub

The components formed by the remaining nested base pairs are extracted as irreducible components by applying the same technique as in the first step. This process is repeated on these extracted components for further extraction until no more new nested components can be found. By collecting all the components being extracted at each recursion level, the whole secondary structure is decomposed into primitive components.

The last step is to evaluate the genus for each of these primitive components. Every primitive component is transformed to a graph identical to that presented in Figure 2 of [20]. Let_
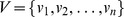
_ be the set of vertices for the n paired nucleotides in the component. Define 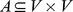
_ as_ the set of arcs representing the base pairs in a bi-directional manner, i.e. for vertices 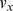 and 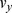 representing two nucleotides of a pair, 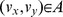 and 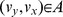. Let 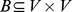 be the set of edges along the backbone from 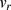 to 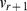, i.e. 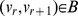 where 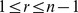. The structure diagram is then represented by the graph 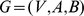. The following pseudocode presents the path traversal in 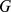 to determine the number of closed loops, 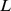.

num_of_closed_loop  = 0;

v  =  vertex in V with an unvisited outgoing arc;

while v exists

a  =  outgoing arc of v;

if a is visited

num_of_closed_loop++;

v  =  vertex with an unvisited outgoing arc;

else

v’  =  vertex from v via a;

mark a as visited;

if there exists an unvisited outgoing edge b of v’

v  =  vertex from v’ via b;

mark b as visited;

else

v  =  vertex with an unvisited outgoing arc;

end if

end if

end while

Together with the number of base pairs involved, 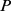, the genus value 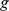 of a particular primitive component can be evaluated.

We are also interested to know that, given a genus 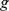, the population of distinct primitive pseudoknot motifs whose genera are equal to 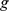. Suppose 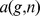 denotes the number of distinct primitive pseudoknots constructed with 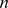 arcs having genus 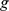, where each arc represents a base pair stem (not a base pair). Given that a primitive pseudoknot with genus 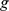 must be constructed within 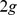 to 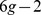 arcs [21], the number of distinct primitive pseudoknot with genus 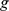, 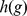, is given by:
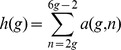



To evaluate 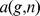, we devise a novel top-down tree building algorithm to enumerate all possible structures with a given value of 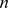. Every leaf node of the tree consists of a distinct primitive pseudoknot expressed by a pseudoknot pattern token, such as “ABACBC” which denotes a primitive pseudoknot (kissing hairpin) constructed with 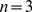 base pair arcs, with each arc represented by a pair of identical character. In the kissing hairpin example, the arc represented by a pair of “A” crosses the left end of the arc represented by the “B” pair, while the arc represented by the “C” pair crosses its other end. Each node has a first-in-first-out (FIFO) arc character queue for left end, and a character set for right end, as well as a pseudoknot pattern token. The root level is level zero and so the leaf level is 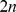. A new left end character is enqueued to the left end queue of each node prior to child node creation when the tree level is less than 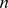. Starting from the root node, when child nodes, which are of the same level, are built, they extend all possible primitive pseudoknot patterns by each node appending one of the available arc characters to its own pseudoknot pattern token. For any non-leaf node, if the left end character queue is non-empty, then the first character will be dequeued for its child node creation, and this character will be added to the right end character set later. For the right end character set, each character is selected once to create a child node if conditions are met, and this character is then eliminated from the right end character set of that child node. Every non-root node inherits a pseudoknot pattern token, left end character queue and right end character set from its parent node. At the leaf node level, all pseudoknot pattern tokens are completed and their genus values are then evaluated. The following three rules restrict the generation of child nodes from any non-leaf node:

*Rule 1*: To avoid reducible or nested pseudoknot-free substructures, when a new character is selected at one level as left end, it is only available for selection for right end after the node at the next (child) level has been generated.

*Rule 2*: To avoid reducible or nested pseudoknots, before a non-leaf child node is generated, it is checked to guarantee that the selected character does not result in reducible or nested pseudoknots in the whole pattern token.

*Rule 3*: To avoid collapsible arcs, if a character Y is selected as the left end at the child node of its parent node which, in turn selects character X as its left end, then later when a descendant node selects character X as its right end, character Y will not be available for selection for its child node. In other words, a pattern …YX….XY… is avoided in final pseudoknot pattern tokens.

The above three rules were incorporated into the tree building algorithm as shown below:

N  =  number of arcs from input;

leaf_level  = 2 * N;

root_node  =  initialize with empty left end character queue, right

end character set and pseudoknot pattern token, level  = 0;

enqueue a new character to left end character queue of root_node;

generate_child_nodes(root_node);

sub generate_child_nodes (node)

//Create a child node and append the first left end character to

//its token if the respective queue is not empty

if left end character queue of node is not empty

child_node  =  copy of node;

s  =  dequeued character from left end character queue of

child_node;

append s to pseudoknot pattern token of child_node;

child_node->level++;

if child_node->level < N

enqueue a new character to left end character queue of

child_node;

end if

//Enforce Rule 1

if there exists a preceding left end character w appended to

pseudoknot pattern token of node at level  =  =  node->level –1

add w to right end character set of child_node;

end if

generate_child_nodes(child_node);

end if

//Create child node(s) for every right end character in the set if

//the rules are not violated

for each character e in right end character set

//Enforce Rule 2

if node–>level ≠ leaf_level and e gives reducible or nested

substructure when appended to pseudoknot pattern token

next;

end if

//Enforce Rule 3

if e gives collapsible arcs when appended to pseudoknot pattern

token

next;

end if

child_node  =  copy of node;

append e to pseudoknot pattern token of child_node;

remove e from right end character set of child_node;

child_node->level++;

if child_node->level  =  =  leaf_level

//Primitive pseudoknot is completed at leaf node level

report pseudoknot pattern token of child node, evaluate the

genus value of the pseudoknot it represents and increment

respective count for genus accordingly;

else

//Enforce Rule 1

if there exists a preceding left end character w appended to

pseudoknot pattern token of node at level  =  =  node->level –1

add w to right end character set of child_node;

end if

generate_child_nodes(child_node);

end if

end for

end sub

**Figure 3:**
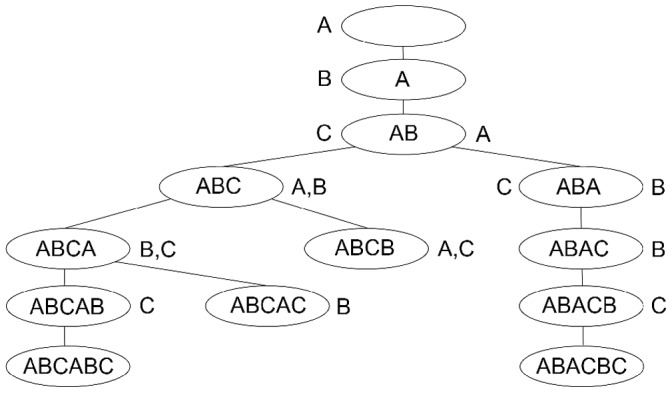
Primitive pseudoknot tree constructed with 3 arcs. Characters on the left of any internal node belong to the FIFO left end character queue, while those on its right belong to the right end character set. Nodes with pseudoknot pattern token ABCB cannot be extended because appending the right end character A violates rule 3, while appending character C violates rule 2. Similarly, for nodes with token ABCAC, appending character B also violates rule 3. At the lowest leaf node level, we obtain tokens ABCABC (pseudotrefoil) and ABACBC (kissing hairpin).


Figure 3 illustrates the generation of the two primitive pseudoknots constructed with 3 arcs, which are the kissing hairpin and pseudotrefoil. A depth first approach is adopted for the recursion of child node generation. Once a leaf node is generated, its pattern token is evaluated immediately for the genus value, which increments the count for the appropriate 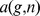. It is then disposed of and the algorithm continues at the last branching node. Hence, the space complexity is O(n). Although the time complexity of the algorithm is exponential, results can still be achieved with 12 arcs. Later, we will see that this method already covers most of the primitive pseudoknots in our dataset. To validate the correctness of the algorithm, we transform the values obtained into the polynomial:
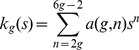



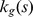
 lists the counts of distinct primitive pseudoknots with genus 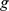 grouped by 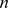 constituent arcs. A pseudoknot-free primitive structure (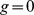), which is either an empty structure (i.e. zero arc) or a single arc structure is represented by:
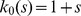



The summation of 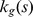 for all values of g gives the power series 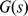:
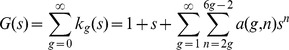




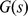
 gives the counts of distinct primitive components with 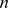 constituent arcs. This power series must be identical to that deduced in [22].

**Table 1:** Composition of the experimental dataset obtained from RNA STRAND.

Source database	RNA types in sourcedatabase	Secondary structure determinationapproach	No. of sequencesin dataset
RCSB Protein Data Bank (PDB)	Ribozymes, ribosomal RNA, transfer RNA, etc.	NMR, X-ray crystallography with RNAView as secondary structure annotation tool	49
Comparative RNA web site	Ribosomal RNA and intronicRNA	Comparative sequence analysis	821
tmRNA database	Transfer messenger RNA	Comparative sequence analysis	645
RNase P database	Ribonuclease P RNA	Comparative sequence analysis	395
Rfam database	Ribozyme, telomerase RNA,RNase MRP RNA,RNase 5’ UTR	Comparative sequence analysis, phylogenetic analysis, mfold, etc.	29

After knowing the population size of primitive pseudoknots with a specified number of stems and genus, real RNA data is then analysed with the topological classification described above to classify the preferred structures in that population. A more abundant RNA database, RNA STRAND (version 2.0) [23], is selected as the data source for analysis rather than PseudoBase [24] as it contains almost 2000 RNA pseudoknotted structure records compared to 359 records in PseudoBase at the time of writing. RNA STRAND is a collaboration of a variety of RNA databases such as RCSB PDB for ribozymes, ribosomal RNAs, transfer RNAs [25], tmRNA database for transfer messenger RNAs [26], Rfam Database for hammerhead ribozymes, telomerase RNAs, RNase MRP RNAs [27], etc. Therefore, our analysis can consider diverse RNA functional categories. Meanwhile, the accuracy of the RNA secondary structures is guaranteed as these structures were determined through comparative sequence analysis or annotation from 3D structures obtained by NMR or X-ray crystallography through RNAView [28].

**Table:** **Table 2.** Values of 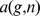 for 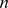 value up to 12.

a(g,n)	2	3	4	5	6	7	8	9	10	11	12
**1**	1	2	1								
**2**			17	160	566	1004	961	476	96		
**3**					1259	23482	176303	727936	1868651	3156754	3584897
**4**							200589	5850396	70846128	487848188	2176440862
**5**									54766516	2229417608	38374062358
**6**											22839203295

The experimental dataset was obtained from RNA STRAND by selecting non-redundant single pseudoknotted RNA molecules. In RNA STRAND, there are cases where different structures exist for a single RNA base sequence. These structures are regarded as redundant and so only one of them was selected arbitrarily. Therefore, the sequences in the dataset are distinct in a pairwise manner. Moreover, some compounds, such as ribosome, may contain an RNA complex rather than a single RNA molecule, and they were not selected. The dataset downloaded from the RNA STRAND web site contained 1957 RNA sequences according to the above criteria. Data pre-processing was carried out to remove non-canonical base pairs in RNA samples prior to importing them into a database table. 18 records were found containing no pseudoknots after pre-processing which were discarded, leaving 1939 sequences. The sequences were not filtered by a certain sequence similarity threshold, the reason being that RNA molecules can be more conserved in structure than in sequence [29], making it hard to define an appropriate threshold. Later, we will also see that some molecules showing similar structures come from different phylogenetic domains or organelles. Table 1 lists the composition of the dataset used in our analysis. Some source databases such as SRP Database and Sprinzl tRNA Database do not appear in the dataset, because no pseudoknots were found in the SRP RNA and tRNA molecules provided.

**Table 3:** Overall statistics of topologically classified primitive pseudoknots.

Genus	Subclass	Pseudoknot pattern	No. of RNA sequences with this primitive pseudoknot	Avg. length±std.dev. (nts)	Min. length (nts)
1	1A	ABAB	1688	204.6±294.9	17
1	1B	ABACBC	365	253.6±282.5	40
1	1C	ABCABC	51	73.6±43.9	37
2	2A	ABCACDBD	45	53.3±0.5	53
2	2B	ABCDCADB	5	71.4±1.2	69
2	2C	ABCDEDBCAE	2	347.5±5.5	342
2	2D	ABCBDEDCAFEF	1	1452±0	1452
3	3A	ABACDEFGEHGIJIFHKBKDJC	48	2637.4±181.2	2268
3	3B	ABCDEFDGFHIHEGJAJCIB	5	2742.8±408.4	2294
3	3C	ABACDEDFGFECHBHIGI	5	2652.6±12.7	2643
3	3D	ABACDEFDGFHIHEGJBJIC	1	2548±0	2548
4	4A	ABCDEFEGCGHIAIJKBKFJHD	1	369±0	369
4	4B	ABACDEFGHEGIHJKJFILBLDKC	1	2617±0	2617
5	5A	ABCDEFEGHCHIJIGKALJLMNBNFMKD	2	431±0	431

The length is measured as the end-to-end distance between (and including) the leftmost base paired nucleotide and the rightmost base paired nucleotide.

**Table 4:** RNA sequence Ids (in RNA STRAND) and RNA family for subclasses with genus ≥2.

Subclass	RNA sequence Ids	RNA family (Organism)
2A	CRW_00467–469, CRW_00471, CRW_00472, CRW_00474–480, CRW_00482–492,CRW_00494–496, CRW_00498, CRW_00499, CRW_00501–503, CRW_00506–509,CRW_00511, CRW_00512, CRW_00515–518, CRW_00520, CRW_00521,CRW_00547, CRW_01456	23S Ribosomal RNA
2B	PDB_00335, PDB_00714, PDB_00716, PDB_00764, PDB_00765	Ribozyme (*Hepatitis Delta Virus*)
2C	ASE_00194, ASE_00204	Ribonuclease P RNA (*Mycoplasma Pneumonia*)
2D	PDB_00187	23S Ribosomal RNA (*Deinococcus Radiodurans*)
3A	CRW_00471–474, CRW_00476–492, CRW_00494–496, CRW_00498–503, CRW_00506–513, CRW_00515–518, CRW_00520, CRW_00522, CRW_00529, CRW_00544, CRW_00547, CRW_01456	23S Ribosomal RNA
3B	CRW_00505, CRW_00523, CRW_00527, CRW_00528, CRW_00546	23S Ribosomal RNA
3C	PDB_00447, PDB_00606, PDB_00628, PDB_00993, PDB_01184	23S Ribosomal RNA
3D	CRW_00525	23S Ribosomal RNA (*Giardia Intestinalis*)
4A	CRW_00001	Group II Intron (*Saccharomyces Cerevisiae*)
4B	CRW_00521	23S Ribosomal RNA (*Euglena Gracilis*)
5A	CRW_00002, CRW_00575	Group II Intron (*Saccharomyces Cerevisiae*)

Consecutive sequence IDs are presented by a range, e.g. CRW_00501–503 represents IDs CRW_00501, CRW_00502 and CRW_00503. Organism names are also given if all molecules belong to a single organism.

In previous topological classification analysis, each base pair stem was converted to a single arc (effective base pair) for more efficient genus evaluation [20]. However, since we are interested in primitive components representing actual secondary structures, this process was omitted. We extracted irreducible components from the RNA secondary structures, and then obtained primitive components by further extracting nested components. Finally, we evaluated the genus values of the primitive pseudoknotted components.

**Figure 4:**
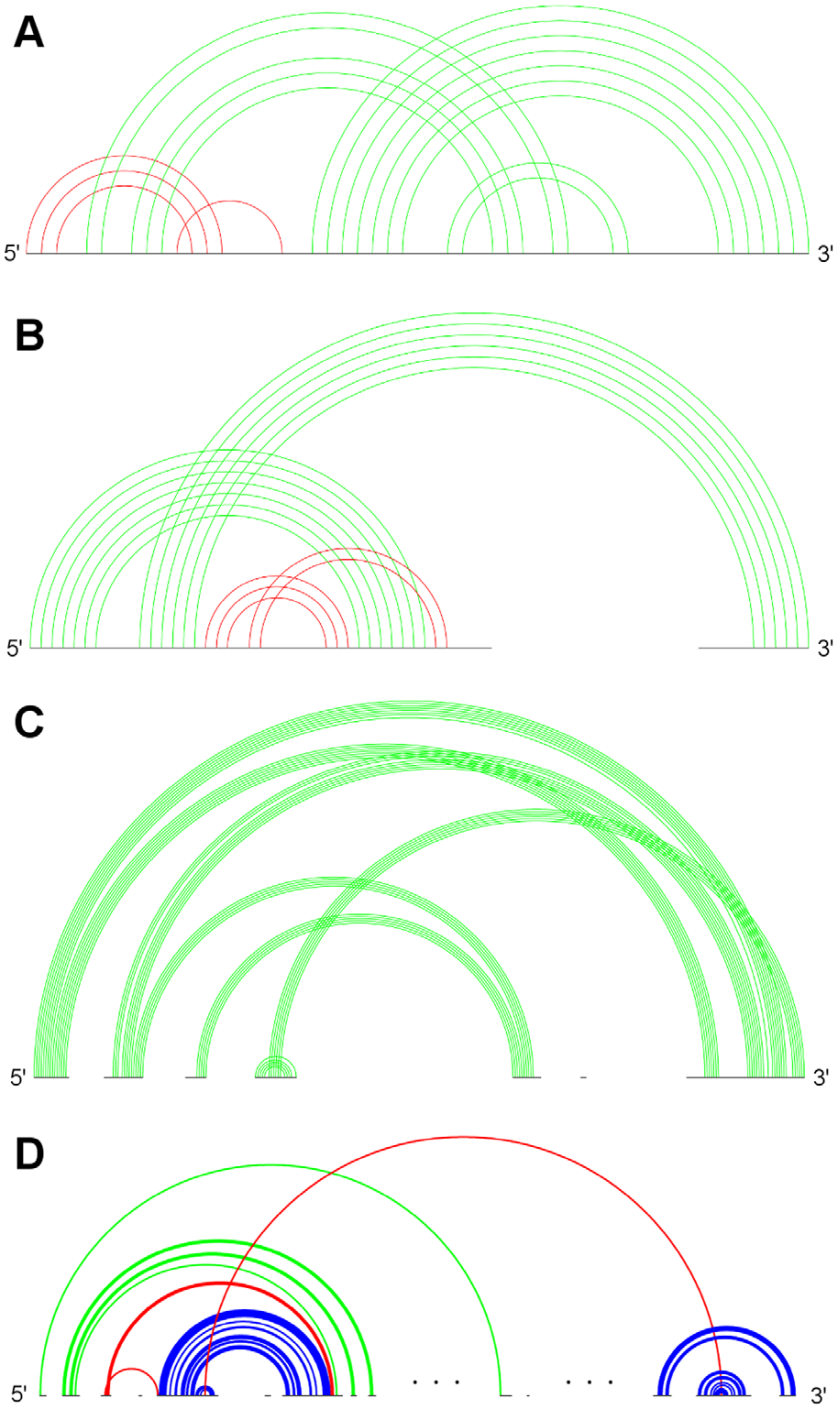
Typical sample structures for all genus 2 subclasses . A: RNA sequence CRW_00474 of subclass 2A with pseudoknot pattern ABCACDBD. B: RNA sequence PDB_00335 of subclass 2B with pseudoknot pattern ABCDCADB. C: RNA sequence ASE_00204 of subclass 2C with pseudoknot pattern ABCDEDBCAE. D: RNA sequence PDB_00187 of subclass 2D with pseudoknot pattern ABCBDEDCAFEF. (Stem color, blue: major stem; green: intermediate stem; red: minor stem).

**Figure 5:**
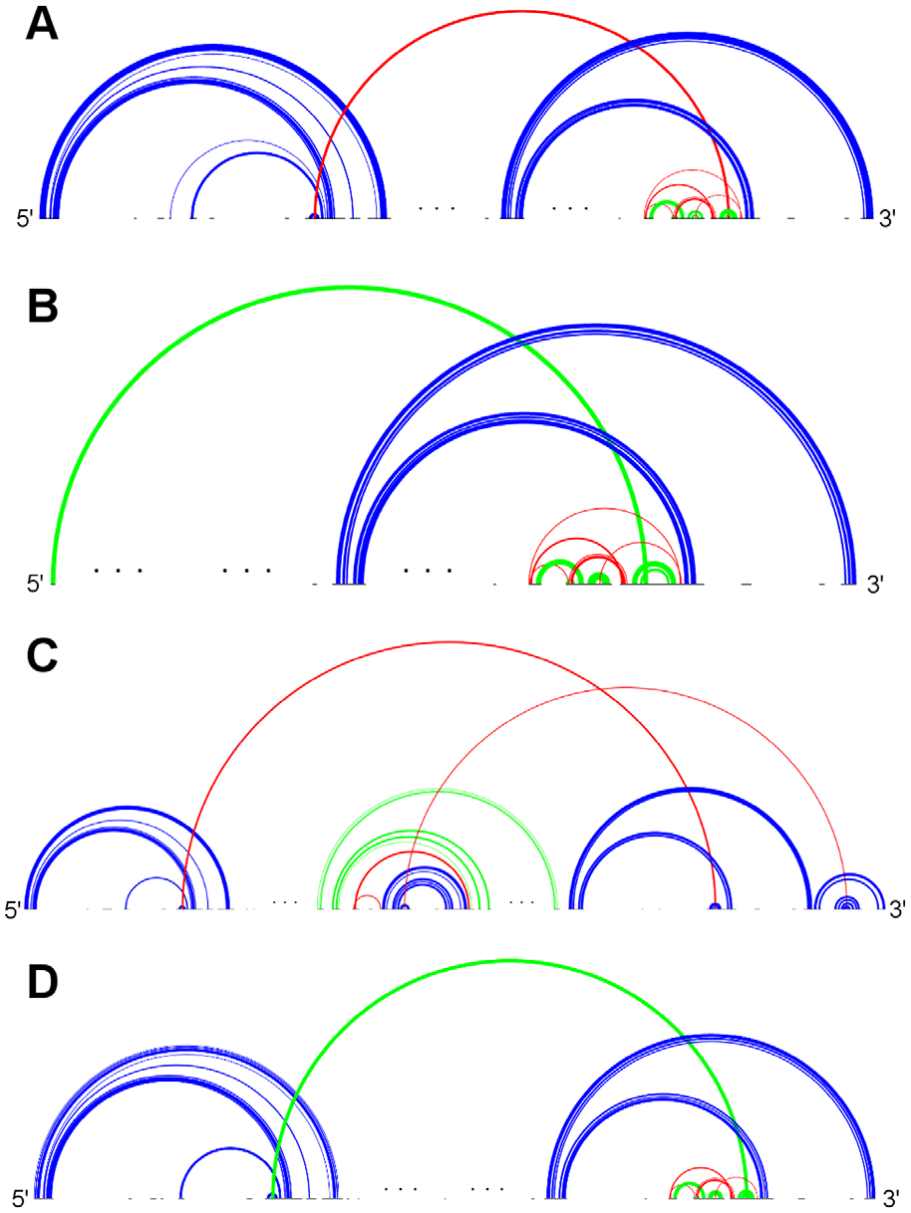
Typical sample structures for all genus 3 subclasses . A: RNA sequence CRW_00490 of subclass 3A with pseudoknot pattern ABACDEFGEHGIJIFHKBKDJC. B: RNA sequence CRW_00527 of subclass 3B with pseudoknot pattern ABCDEFDGFHIHEGJAJCIB. C: RNA sequence PDB_00628 of subclass 3C with pseudoknot pattern ABACDEDFGFECHBHIGI. D: RNA sequence CRW_00525 of subclass 3D with pseudoknot pattern ABACDEFDGFHIHEGJBJIC. (Stem color, blue: major stem; green: intermediate stem; red: minor stem).

**Figure 6:**
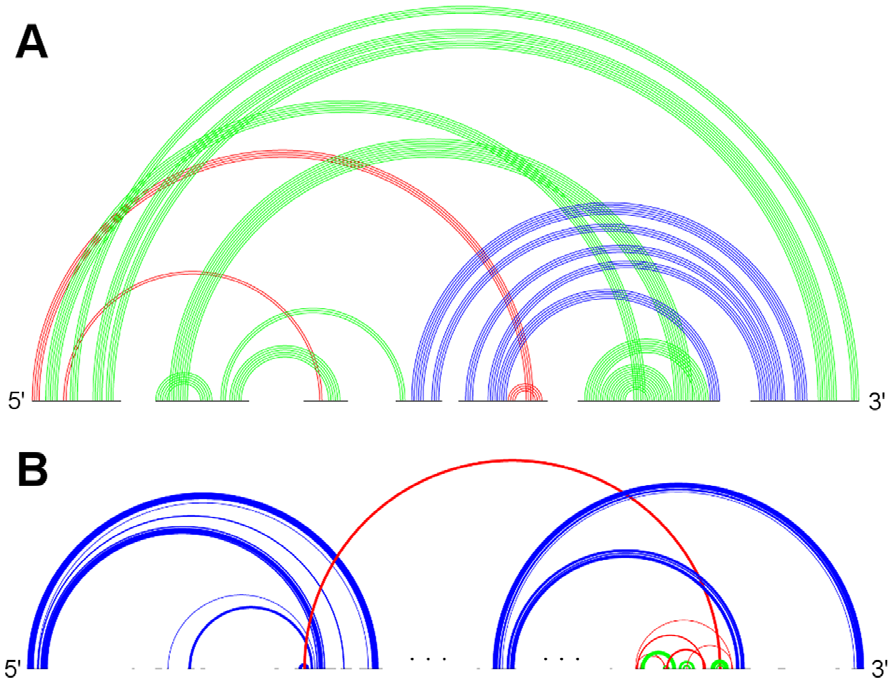
Typical sample structures for all genus 4 subclasses . A: RNA sequence CRW_00001 of subclass 4A with pseudoknot pattern ABCDEFEGCGHIAIJKBKFJHD. B: RNA sequence CRW_00521 of subclass 4B with pseudoknot pattern ABACDEFGHEGIHJKJFILBLDKC. (Stem color, blue: major stem; green: intermediate stem; red: minor stem).

**Figure 7:**
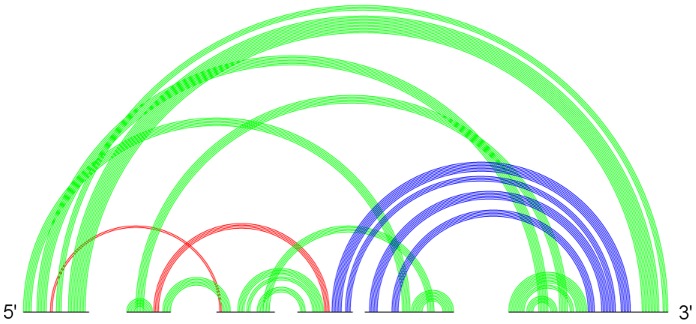
Typical sample structures for all genus 5 subclasses . RNA sequence CRW_00575 of subclass 5A with pseudoknot pattern ABCDEFEGHCHIJIGKALJLMNBNFMKD. (Stem color, blue: major stem; green: intermediate stem; red: minor stem).

The resulting primitive components and their genera were stored in a MySQL database for analysis.

**Table 5:** Comparison of genus of the whole secondary structure between RNA sequences in the PDB dataset (190 sequences in total) and in the RNA STRAND dataset (1939 sequences in total).

Total genus	No. of RNA sequences reportedin PDB dataset	Proportion	No. of RNA sequences reported inRNA STRAND dataset	Proportion
1	66	0.347	597	0.308
2	23	0.121	263	0.136
3	5	0.026	414	0.214
4	18	0.095	585	0.302
5	8	0.042	22	0.011
6	2	0.011	0	0
7	3	0.016	7	0.004
8	2	0.011	2	0.001
9	2	0.011	8	0.004
10	5	0.026	40	0.021
11	4	0.021	1	0.001
12	9	0.047	0	0
13	3	0.016	0	0
14	34	0.179	0	0
15	4	0.021	0	0
16	0	0	0	0
17	1	0.005	0	0
18	1	0.005	0	0

## Results

Table 2 shows the values of 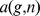 up to 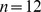. Using these values, the polynomials can be formed as:
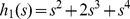


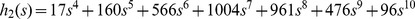



The first 13 terms of 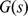
can be evaluated:
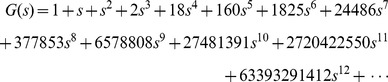



The above power series is identical to that deduced in [22] (equation (11)). This shows the correctness of the tree building algorithm.

**Figure 8:**
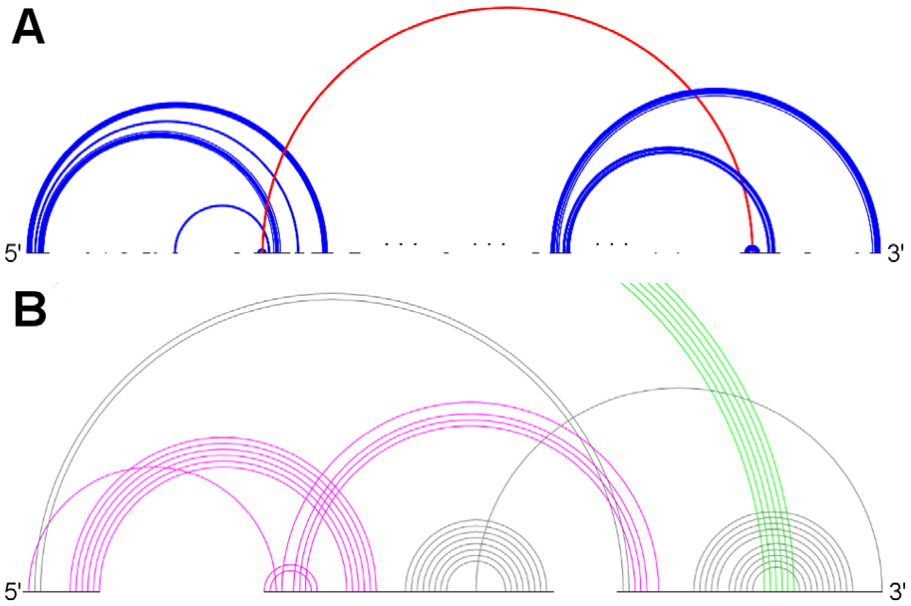
Primitive large kissing hairpin and the G-ribo ring interacting with the large kissing hairpin backbone. A: Large kissing hairpin from RNA sequence PDB_00029 similar to the kissing hairpin backbone. (Stem color, blue: major stem; red: minor stem) B: G-ribo ring (magenta) is crossed with kissing stem (green) through the lone pair and the double pair in CRW_00525 (subclass 3D).

**Figure 9:**
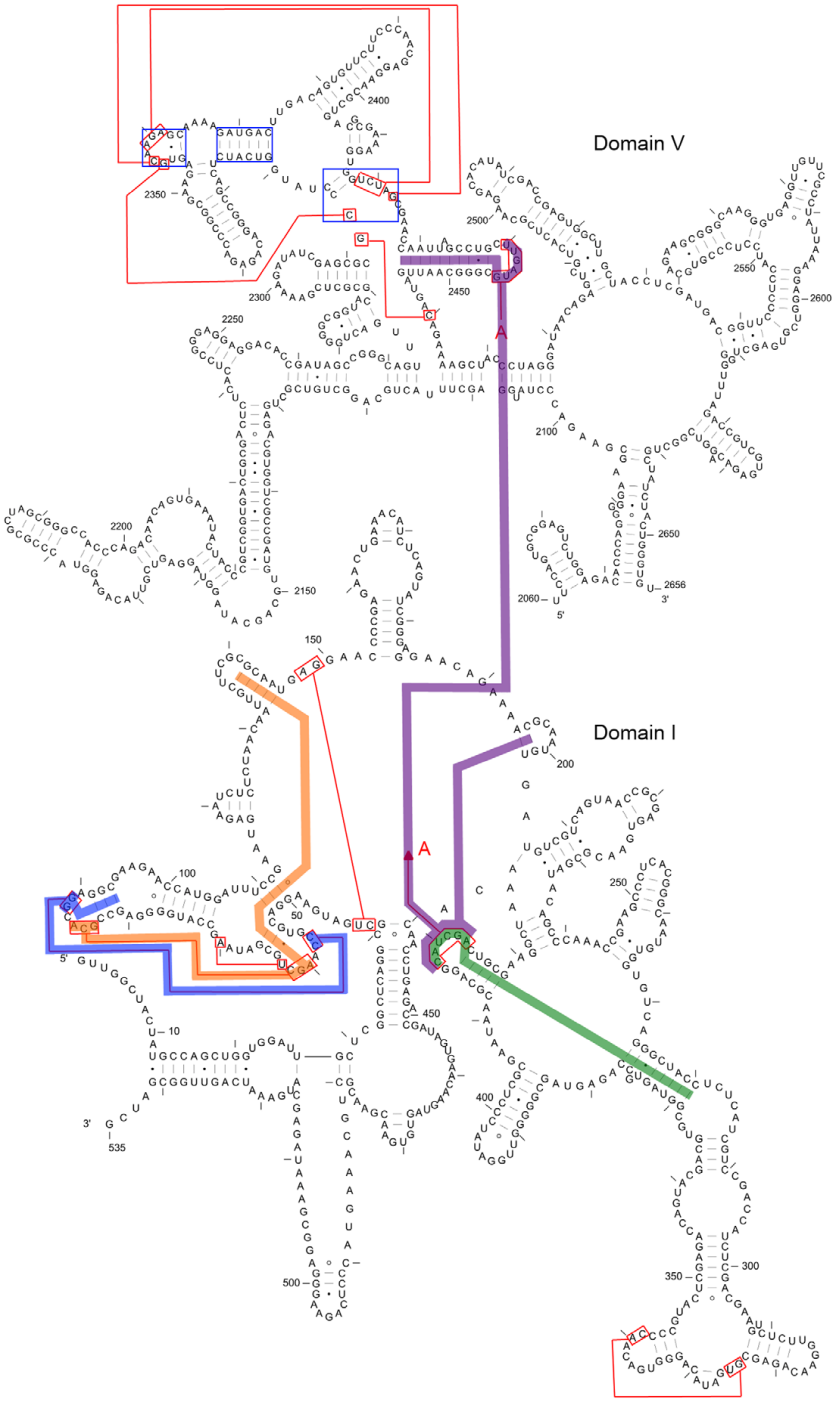
Relevant COIN stacks for the complex pseudoknots in *Haloarcula marismortui* (RNA STRAND Id: CRW_00467). The magenta line represents an interdomain COIN stack and the green line represents an intradomain COIN stack. They exist together in the large kissing hairpin motif. The COIN stacks represented by orange and blue lines exist in the subclass 2A complex pseudoknot. Also, the base pairs in the G-ribo ring motif are marked within the blue squares. For simplicity some other base pairs crossing different domains are not shown.

**Figure 10:**
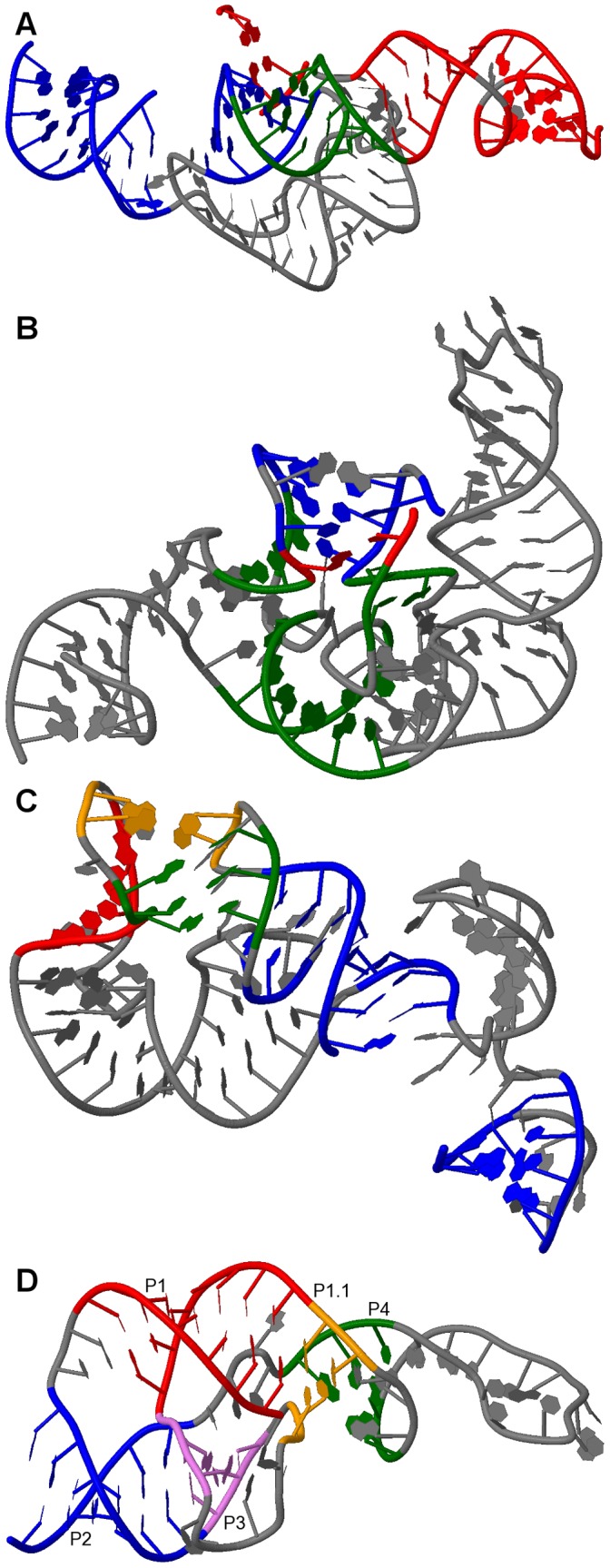
3D structures for special pseudoknot motifs. A-C were derived from RNA molecule 1S72 (*Haloarcula marismortui)* of PDB, and D was derived from 1VC6 (*Hepatitis Delta Virus*). A: Kissing site of the large kissing hairpin. The stems for the interdomain COIN stack are in red and those for intradomain COIN stack are in blue, while the kissing stem is in green. B: G-ribo ring. The lone pair in the motif is in red and another 4-base pair hairpin stem is in blue. The kissing stem is in green. C: COIN stacks in subclass 2A complex pseudoknot. The base pair stems in red and yellow form one intradomain COIN stack while those in blue and green form another. The yellow and green stems together become a single kissing stem. D: Double pseudoknot motif. Stems P1 (red), P1.1 (yellow) and P4 (green) together comprise one stack, while stems P2 (blue) and P3 (pink) form another stack.

On the other hand, in our secondary structure analysis, all the structures were transformed to primitive components and the pseudoknot motifs were classified according to their genera. Table 3 summarizes the overall statistics of different topology classes for these primitive pseudoknots of the 1939 RNA secondary structures. In the table, each primitive pseudoknot pattern is assigned to a subclass. For example, the H-pseudoknot is subclass 1A. The highest genus of a primitive component is 5 in the RNA STRAND dataset, consisting of 14 arcs (stems). Therefore, our primitive structure enumeration algorithm can cover most of the naturally occurring primitive pseudoknots. Table 4 provides the IDs of the RNA sequences in each subclass with genus 2 or higher, and the RNA family, or more specifically, the organism, these sequences pertain to. Some subclasses contained RNA sequences from different phylogenetic domains or organelles. For example, in subclass 2A, RNA sequence CRW_00471 belongs to bacteria and CRW_01456 belongs to cyanelle. In subclass 3A, besides CRW_00471 in bacteria, sequence CRW_00529 belongs to eukaryotes and sequence CRW_00544 belongs to mitochondrion.

We first illustrate how only a few complex pseudoknots were discovered in nature when compared with the population of theoretical candidates. From Table 3, both subclasses 2A and 2B represent two distinct pseudoknot motifs, both constructed with 4 stems with the same classified complexity. From Table 2, we know that the other 15 candidates are still not found. The contrast is more apparent when we consider higher genus motifs. Subclass 4A represents a genus 4 pseudoknot motif constructed with 11 stems, but it is just one out of over 480 million possibilities.

Figures 4, 5, 6, and 7 depict the representative structures for all pseudoknot motifs discovered in the analysis and whose genera are higher than one. For the rest of this paper, we denote a base pair stem with at least 20 base pairs as a major stem, a stem with more than at least 5 base pairs and less than 20 base pairs as an intermediate stem, and a stem with less than 5 base pairs as a minor stem. In the figures, these major, intermediate and minor stems are coloured in blue, green and red, respectively. Furthermore, the gaps breaking the backbone (represented by the black horizontal line) means nested substructures at those gaps that are extracted during analysis. And the symbol “…” represents a long gap or a long backbone with unpaired nucleotides.

When compared to the primitive pseudoknot genus distribution of the PDB dataset in [20], our dataset also follows the same trend with genus 1 primitive pseudoknots dominating (5062 counts), and the distribution counts decrease with higher genus values. A comparison was also performed in the total genus of the whole RNA secondary structure, as shown in Table 5. The two most significant differences between both datasets are the large discrepancy in the highest total genus evaluated and the different total genus distribution. In the PDB dataset, the highest genus reported is 18 while in the RNA STRAND dataset, it is only 11; and the highest genus of the primitive pseudoknot is 13 in the PDB dataset compared to 5 in our data. Due to different selection criteria (non-redundant single molecule), not all RNA sequences in the PDB dataset were covered in our dataset, especially those molecules in the ribosomal RNA complex. Therefore, we prepared another set of RNA sequences from the RNA STRAND database that consists of all sequences in the PDB dataset for direct comparison (called the STRAND-PDB dataset). It was found that our evaluated values were usually lower even though we did not remove the non-canonical base pairs and considered the multiple molecules in the RNA complex as a single molecule (data not shown). The most complex primitive component and secondary structure have genera of 5 and 11, respectively which is equal to our original dataset. We account for this by the different secondary structure annotation used. RNAView was used for annotation in RNA STRAND, but we do not know what annotation was used in the PDB dataset. Nevertheless, the total genus values of a particular molecule evaluated from the PDB dataset and the STRAND-PDB dataset are highly correlated, as indicated by the Pearson correlation coefficient of 0.93. For a different total genus distribution, from Table 5 we found that the proportions of genus 3 and genus 4 structures are much higher compared to the PDB dataset. According to Table 3, there are only 61 sequences consisting of primitive pseudoknots of genera 3 or 4, and so there are many genera 3 or 4 RNA molecules assembled with lower genus pseudoknots. This is not reflected in the PDB dataset.

## Discussion

In the results section, we have seen that only a few primitive pseudoknots with genus higher than 1 were found in the RNA STRAND dataset. Now we are going to identify some common structural features, if any, and their contribution to the overall stability whenever possible. According to the representative structures illustrated in Figures 4, 5, 6, and 7, we observed some similarities between different subclasses.

It was observed that, from Figures 4, 5, and 6, some motifs are formed by a large kissing hairpin with its kissing stem crossing with two minor stems of a local pseudoknot inside the hairpin loop near to the 3′ end. We call this large kissing hairpin the “kissing hairpin backbone” of the pseudoknot motif. Structurally, it consists of two major stem hairpins with their loops interacting with each other. All the instances of subclasses 2D, 3A, 3D and 4B possess such a backbone in their structures. Subclass 3C even represents a motif of which the backbone is an interlock of two large kissing hairpins. All these subclasses belong to the 23S Ribosomal RNA family. We discovered that, in this family, there are another five RNA molecules (CRW_00467, CRW_00468, CRW_00469, CRW_00475, PDB_00029) consisting of a large kissing hairpin (over 2500 nucleotides, belonging to subclass 1B) which is structurally highly similar to the backbone structure identified in those subclasses except subclass 2D, as depicted in Figure 8A. This kissing interaction is involved in the continuous interhelical base stacking (COIN stacking) found in large RNA structures [30]. COIN stacking is a particular type of base stacking occurring between two or more helices. This tertiary interaction brings a stabilization effect to the overall structure. The COIN stacks of the 23S ribosomal RNA molecule *Haloarcula marismortui* have been annotated [30], [31] and Figure 9 shows the relevant COIN stacks. Several base pair stems including the kissing stem of the large kissing hairpin motifs form a COIN stack according to [30], and they are connected by the magenta line in the figure. This stack is an interdomain COIN stack as it spans across domains I and V in 23S ribosomal RNA. The two domains were brought close to each other by this interdomain stack in such a way that particular stems in each domain are stacked together along with the kissing stem, as shown in Figure 10A. Although the other 23S ribosomal RNA molecules are not annotated, we believe that in those conserved large kissing hairpins (or backbones) of the other 23S RNA molecules, this interaction stacking with the kissing stem also occurs. In Figure 9, there is another COIN stack (shown by green line) also involving the same kissing stem. As a result, these two COIN stacks included the kissing stems as well as the two hairpin stems. This configuration favours the long hairpin stem because of the stronger stacking effect, so we observed the major stems at both hairpins in most cases. However, we have to emphasize that the kissing hairpin is not a prerequisite for COIN stack formation. There are many COIN stacks in *Haloarcula marismortui* that do not involve any kissing stem [30]. Therefore, such stacking is anticipated to be found in the “incomplete” kissing hairpin backbone (the left hairpin is absent) subclass 3B. We believe that COIN stacks substantially stabilize structure.

The main difference between motifs possessing the kissing hairpin backbone and the large kissing hairpin is the local pseudoknot that crosses with the kissing stem near the 3′ end. This pseudoknot is essentially a G-ribo ring [32]. Figure 8B illustrates the G-ribo ring and the two lone pairs where the first pair crosses both this motif and the kissing stem, while the second pair crosses the first lone pair. This G-ribo ring does not always interact with the large kissing hairpin. It also exists in molecules containing the large kissing hairpin except molecule PDB_00029. The quasicoaxial stacking of helical stems stabilizes the G-ribo ring [32], but whether the interaction between this pseudoknot and the kissing hairpin backbone further stabilizes the complex pseudoknot or not is undetermined. Figure 9 and Figure 10B show the location and the typical structure of a G-ribo ring, respectively.

Topological classification identified another complex pseudoknot motif which is also from 23S ribosomal RNA molecules, as represented by subclass 2A. This motif involves two distinct COIN stacks illustrated by orange and blue lines in Figure 9. Both stacks include different parts of the kissing stem, which are separated by an unpaired nucleotide in each kissing loop. Moreover, these two stacks only consist of stems in domain I and are so called intradomain COIN stacks. All RNA molecules in this subclass have a lone pair constituting a stem. In the analysis of the STRAND dataset, this lone pair does not appear in every 23S ribosomal molecule, and in the absence of this base pair, a kissing hairpin appears instead. The lone pair was found in *Haloarcula marismortui* from our dataset (U63-A70 pair) but not in the structure provided in [30]. Nevertheless, no matter whether the complex pseudoknot or the kissing hairpin appears, the underlying COIN stacks still exist. Figure 10C illustrates the two COIN stacks.

Subclass 2B represents the double pseudoknot motif in *Hepatitis Delta Virus* (HDV) ribozyme [33]. This motif contains a characteristic stem P1.1 consisting of double CG base pairs, which is critical for cleavage activity [34]. There are two coaxial stacks formed, one having stems P1, P1.1 and P4 and the other having stems P2 and P3, as shown in Figure 10D. Therefore, the double pseudoknot motif is stabilized.

We also observed some other large primitive pseudoknotted components whose lengths are comparatively much shorter than those accompanied by the kissing hairpin backbone (usually with lengths over 2000 nucleotides). These pseudoknot motifs consist of mainly intermediate stems, and most of the stems cross several other stems, thus achieving a high complexity within a relatively compact structure. These motifs were found in Group II Introns of *Saccharomyces Cerevisiae* and were classified into subclasses 4A and 5A. They are shown in Figures 6 and 7, respectively. We can also identify a similar structure in Ribonuclease P RNA molecules of *Mycoplasma Pneumonia* visually from Figure 4, and it is classified into subclass 2C. These three subclasses exhibit similar motif lengths (from 342 nucleotides to 431 nucleotides respectively, according to Table 3). However, to the best understanding of the authors, no details concerning these types of pseudoknots are available, and so the underlying interaction that favours their formation is currently unknown, even though it is believed to be related to helical stacking.

Compared to the abundant H-pseudoknot and kissing hairpin as shown in Table 3, other complex primitive pseudoknots (genus ≥2) are far less frequently observed. Their existence usually relies on special helical stacking such as COIN stacking or quasicoaxial stacking that help stabilize the structure. Therefore, we believe that the energy barrier for the formation of complex primitive pseudoknots is much higher than that for the H-pseudoknot and kissing hairpin. This explains why so few complex pseudoknots are discovered in nature. On the other hand, it was reported that coaxial stacking does not occur in some H-pseudoknots [35], implying a lower energy requirement and are thus more prevalent. Another contrast is that since complex pseudoknots usually play an important role in affecting the overall 3D structure and are specific in the functions of the RNA molecules, they are likely to be found in a single RNA family. The existence of the H-pseudoknot and kissing hairpin are, however, not RNA family bound. In particular, there are two special motifs (one represented by subclass 2A and another being the interaction of the large kissing hairpin and the G-ribo ring) belonging to 23S ribosomal RNA family, the latter resulting in pseudoknots of different genus values and structures, thus dominating the subclasses. On the other hand, in other RNA families such as tmRNA and telomerase RNA, only genus 1 pseudoknot motifs were found in our dataset.

From the study of topologically classified primitive pseudoknots, we can see that complex pseudoknots are usually specific well-known motifs or motifs that interact with each other. Their formations are driven by certain helical stacking mechanisms. However, at present, little is known about the thermodynamic aspect of helical stacking, making it hard to parameterize in the MFE model. Therefore, some heuristics are necessary in order to predict complex structures or a particular part of them. For example, current prediction algorithms such as DotKnot can predict the kissing hairpin [36], [37], but the suggested input sequence size is just 400 nucleotides. While this limit is able to cover most of the primitive kissing hairpins found in our dataset, large kissing hairpins or backbone motifs are omitted. To overcome this issue, we suggest that since the major stem occupies both hairpins of kissing interaction, only predicted major stems can be selected as candidates for the formation of the large kissing hairpin or backbone. This allows the algorithms to create appropriate candidates without a severe impact on efficiency. Moreover, the G-ribo ring, which can be regarded as a kissing hairpin, consists of a lone pair as the hairpin stem near the 5’ end, as illustrated in Figure 8B. For efficient computation, lone pairs are usually ignored and so the G-ribo rings are also overlooked. A similar situation also happens to the double pseudoknot motif as the double CG paired stems are also ignored. Future structure prediction approaches should be able to filter lone pairs rather than discarding them all in order to predict these functionally important motifs.

### Conclusion

Topological classification can be utilized to decompose and rank arbitrary pseudoknots according to their complexities which are expressed in terms of a genus value. Following this measure, other than the simplest primitive pseudoknots which include the most prevalent H-pseudoknot and kissing hairpin, complex primitive pseudoknots were discovered existing as some functionally important motifs that were already known. Further tertiary interaction between these motifs may even create variations of complex components, such as the large kissing hairpin crossing with the G-ribo ring in different ways and the resulting motifs have different genera. The energy barrier for these complex pseudoknots is much higher and most of them require helical stacking for stability. This might explain why only a few cases were found in real data despite a large number of possible structures as demonstrated. Still there exists some complex structures of which the reasons for their formations remain unknown, and it is expected that more complex pseudoknots different from those discussed above will be discovered. This classification technique allows us to effectively compare and classify them. Based on the classified results, some suggestions have been proposed to improve the prediction ability for complex pseudoknots.

## References

[1] Storz G (2002). An expanding universe of noncoding RNAs.. Science.

[2] Amaral PP, Dinger ME, Mercer TR, Mattick JS (2008). The eukaryotic genome as an RNA machine.. Science.

[3] Gottesman S (2005). Micros for microbes: non-coding regulatory RNAs in bacteria.. Trends in Genetics.

[4] Repoila F, Majdalani N, Gottesman S (2003). Small non-coding RNAs, co-ordinators of adaptation processes in Escherichia coli: the RpoS paradigm.. Molecular Microbiology.

[5] Lee KS, Varma S, SantaLucia J, Cunningham PR (1997). In vivo determination of RNA structure-function relationships: Analysis of the 790 loop in ribosomal RNA.. Journal of molecular biology.

[6] Chen QF, Chen YPP (2009). Discovery of Structural and Functional Features in RNA Pseudoknots.. Ieee Transactions on Knowledge and Data Engineering.

[7] Tinoco I, Bustamante C (1999). How RNA folds.. Journal of molecular biology.

[8] Zuker M, Stiegler P (1981). Optimal Computer Folding of Large Rna Sequences Using Thermodynamics and Auxiliary Information.. Nucleic acids research.

[9] Rivas E, Eddy SR (1999). A dynamic programming algorithm for RNA structure prediction including pseudoknots.. Journal of molecular biology.

[10] Reeder J, Giegerich R (2004). Design, implementation and evaluation of a practical pseudoknot folding algorithm based on thermodynamics.. BMC bioinformatics.

[11] Theis C, Janssen S, Giegerich R (2010). Prediction of RNA Secondary Structure Including Kissing Hairpin Motifs.. Algorithms in Bioinformatics.

[12] McCaskill JS (1990). The equilibrium partition function and base pair binding probabilities for RNA secondary structure.. Biopolymers.

[13] Dirks RM, Pierce NA (2003). A partition function algorithm for nucleic acid secondary structure including pseudoknots.. Journal of computational chemistry.

[14] Dirks RM, Pierce NA (2004). An algorithm for computing nucleic acid base-pairing probabilities including pseudoknots.. Journal of computational chemistry.

[15] Lyngso RB, Pedersen CN (2000). RNA pseudoknot prediction in energy-based models.. Journal of computational biology : a journal of computational molecular cell biology.

[16] Akutsu T (2000). Dynamic programming algorithms for RNA secondary structure prediction with pseudoknots.. Discrete Applied Mathematics.

[17] Chen WYC, Deng EYP, Du RRX, Stanley RP, Yan CH (2007). Crossings and nestings of matchings and partitions.. Transactions of the American Mathematical Society.

[18] Chen WY, Han HS, Reidys CM (2009). Random K-noncrossing RNA structures.. Proceedings of the National Academy of Sciences of the United States of America.

[19] Jiang M, Tejada PJ, Lasisi RO, Cheng S, Fechser DS (2010). K-partite RNA secondary structures.. Journal of computational biology : a journal of computational molecular cell biology.

[20] Bon M, Vernizzi G, Orland H, Zee A (2008). Topological classification of RNA structures.. Journal of molecular biology.

[21] Reidys CM, Huang FW, Andersen JE, Penner RC, Stadler PF (2011). Topology and prediction of RNA pseudoknots.. Bioinformatics.

[22] Rodland EA (2006). Pseudoknots in RNA secondary structures: representation, enumeration, and prevalence.. Journal of computational biology : a journal of computational molecular cell biology.

[23] Andronescu M, Bereg V, Hoos HH, Condon A (2008). RNA STRAND: the RNA secondary structure and statistical analysis database.. BMC bioinformatics.

[24] van Batenburg FH, Gultyaev AP, Pleij CW (2001). PseudoBase: structural information on RNA pseudoknots.. Nucleic acids research.

[25] Westbrook J, Feng Z, Chen L, Yang H, Berman HM (2003). The Protein Data Bank and structural genomics.. Nucleic acids research.

[26] Andersen ES, Rosenblad MA, Larsen N, Westergaard JC, Burks J (2006). The tmRDB and SRPDB resources.. Nucleic acids research.

[27] Griffiths-Jones S, Moxon S, Marshall M, Khanna A, Eddy SR (2005). Rfam: annotating non-coding RNAs in complete genomes.. Nucleic acids research.

[28] Yang H, Jossinet F, Leontis N, Chen L, Westbrook J (2003). Tools for the automatic identification and classification of RNA base pairs.. Nucleic acids research.

[29] Reiter NJ, Chan CW, Mondragon A (2011). Emerging structural themes in large RNA molecules.. Current opinion in structural biology.

[30] Holbrook SR (2008). Structural principles from large RNAs.. Annual review of biophysics.

[31] Ban N, Nissen P, Hansen J, Moore PB, Steitz TA (2000). The complete atomic structure of the large ribosomal subunit at 2.4 angstrom resolution.. Science.

[32] Steinberg SV, Boutorine YI (2007). G-ribo motif favors the formation of pseudoknots in ribosomal RNA.. Rna-a Publication of the Rna Society.

[33] Ferre-D’Amare AR, Zhou K, Doudna JA (1998). Crystal structure of a hepatitis delta virus ribozyme.. Nature.

[34] Wadkins TS, Perrotta AT, Ferre-D’Amare AR, Doudna JA, Been MD (1999). A nested double pseudoknot is required for self-cleavage activity of both the genomic and antigenomic hepatitis delta virus ribozymes.. Rna-a Publication of the Rna Society.

[35] Cao S, Chen SJ (2006). Predicting RNA pseudoknot folding thermodynamics.. Nucleic acids research.

[36] Sperschneider J, Datta A, Wise MJ (2011). Heuristic RNA pseudoknot prediction including intramolecular kissing hairpins.. Rna-a Publication of the Rna Society.

[37] Chen QF, Chen YPP (2011). Modeling Conserved Structure Patterns for Functional Noncoding RNA.. IEEE Transactions on Biomedical Engineering, 58(6): 1528–1533, 2011.

